# Neurorehabilitation: Neural Plasticity and Functional Recovery 2018

**DOI:** 10.1155/2019/7812148

**Published:** 2019-01-21

**Authors:** Toshiyuki Fujiwara, Junichi Ushiba, Surjo R. Soekadar

**Affiliations:** ^1^Department of Rehabilitation Medicine, Juntendo University Graduate School of Medicine, 2-1-1 Hongo, Bunkyo, Tokyo 113-8421, Japan; ^2^Faculty of Science and Technology, Keio University, 3-14-1 Hiyoshi, Kohoku-ku, Yokohama, Kanagawa 223-8522, Japan; ^3^Keio Institute of Pure and Applied Sciences (KiPAS), Keio University, 3-14-1 Hiyoshi, Kohoku-ku, Yokohama, Kanagawa 223-8522, Japan; ^4^Department of Biosciences and Informatics, Faculty of Science and Technology, Keio University, 3-14-1 Hiyoshi, Kohoku-ku, Yokohama, Kanagawa 223-8522, Japan; ^5^Clinical Neurotechnology Laboratory, Department of Psychiatry and Psychotherapy, Neuroscience Research Center (NWFZ), Charité-University Medicine Berlin, Germany; ^6^Department of Psychiatry and Psychotherapy, Eberhard-Karls-University Tübingen, Germany

Over the last decades, many axiomatic and dominating views on the functional architecture and workings of the mammalian central nervous system (CNS) had to be fundamentally reconsidered. Although the dominating view that the mature mammalian CNS is structurally immutable was repeatedly challenged, e.g., by studies showing collateral axonal sprouting and intracortical synaptic plasticity after a spinal cord injury (SCI) in cats [1, 2], the capacity of the lesioned CNS to reorganize was only fully appreciated after Merzenich and colleagues introduced their famous deafferentation studies in the 1980s [3, 4]. Besides showing that topographic cortical representations are maintained dynamically throughout life, they also provided compelling evidence that this self-organizing capacity of the CNS can relate to neurological recovery [5–7].

Based on this new understanding of CNS plasticity and the factors driving it, Taub et al. introduced a novel rehabilitation procedure that now belongs to the established repertoire of physiotherapists worldwide (Constraint-Induced Movement Therapy, CIMT) [7–9]. Being a good example for the successful translation of insights from basic research findings collected over several decades in animal studies into a new treatment strategy used in hospitals all over the world, the development of CIMT also exemplifies the long, strenuous and often very difficult path from bench to bedside.

This special issue acknowledges this challenging path and provides a forum for presenting the latest views and findings in the field of neurorehabilitation. Besides featuring a comprehensive review on the state-of-the-art in experimental stroke research by A.-S. Wahl (“State-of-the-Art Techniques to Causally Link Neural Plasticity to Functional Recovery in Experimental Stroke Research”) and cognitive rehabilitation in Parkinson's disease by M. Díez-Cirarda et al. (“Neurorehabilitation in Parkinson's Disease: A Critical Review of Cognitive Rehabilitation Effects on Cognition and Brain”), this special issue includes a study by S.-L. Liew et al. that evaluated brain activity during action observation of 24 stroke survivors and 12 age-matched healthy controls using functional magnetic resonance imaging (fMRI) (“Laterality of Poststroke Cortical Motor Activity during Action Observation Is Related to Hemispheric Dominance”). They found that action observation is lateralized to the dominant, rather than ipsilesional, hemisphere. As this may reflect an interaction between the lesioned hemisphere and the dominant hemisphere in driving lateralization of brain activity after stroke, they conclude that this finding should be carefully considered when characterizing poststroke neural activity.

M. R. Pereira-Jorge et al. (“Anatomical and Functional MRI Changes after One Year of Auditory Rehabilitation with Hearing Aids”) describe the anatomical and functional MRI changes related to one year of auditory rehabilitation with hearing aids (HA) across 14 individuals diagnosed with bilateral hearing loss. While they found a reduction in activity in the auditory and language systems and an increase in visual and frontal cortical areas, the use of HA over one year increase the activity in the auditory and language cortices as well as multimodal integration areas. Moreover, they found an increased cortical thickness in multimodal integration areas, particularly the very caudal end of the superior temporal sulcus, the angular gyrus, and the insula. P. Álvarez Merino et al. (“Evidence Linking Brain Activity Modulation to Age and to Deductive Training”) investigated the effect of deductive reasoning training on modulation of electric brain activity and compared this modulation between younger (mean age 21 ± 3.39 years) and older (mean age 68.92 ± 5.72 years) healthy adults. While younger adults showed symmetric bilateral activity in anterior brain areas in their study, older adults showed asymmetrical activity in anterior and posterior brain areas. They conclude that bilateral brain activity modulation may be an age-dependent mechanisms to maintain cognitive function under high demand.

To better understand the role of serotonergic receptors for functional recovery after SCI, K. Miazga et al. analyzed the mRNA of serotonergic 5-HT2A and 5-HT7 receptors (encoded by Htr2a and Htr7 genes) in motoneurons of rats with and without SCI (“Intraspinal Grafting of Serotonergic Neurons Modifies Expression of Genes Important for Functional Recovery in Paraplegic Rats”). They found that intraspinal grafting of serotonergic neurons can modify the expression of Htr2a and Htr7 genes suggesting that upregulation of these genes might account for the improved locomotion found after intraspinal grafting.

Based on a number of studies suggesting a neuroprotective effect of green tea (*Camellia sinensis*), P. M. Sosa et al. investigated whether green tea and red tea have a comparable effect on motor deficits and striatum oxidative damage in rats with hemorrhagic stroke (“Green Tea and Red Tea from *Camellia sinensis* Partially Prevented the Motor Deficits and Striatal Oxidative Damage Induced by Hemorrhagic Stroke in Rats”). They found that the two teas seemed equally effective.

M S. Sherwood et al. evaluated resting cerebral perfusion before and after transcranial direct current stimulation (tDCS), a form of transcranial electric stimulation (tES), applied to the left prefrontal cortex to investigate the underlying neural mechanisms of tDCS on cognitive brain functions (“Repetitive Transcranial Electrical Stimulation Induces Quantified Changes in Resting Cerebral Perfusion Measured from Arterial Spin Labeling”). They found that tDCS increased cerebral perfusion across many areas of the brain as compared to sham stimulation. As this effect originated in the locus coeruleus linked to the noradrenergic system, the authors suggest that the broad behavioral effects of frontal lobe tDCS might relate to a modulation of the locus coeruleus that excites the noradrenergic system.

S. Betti et al. (“Testing rTMS-Induced Neuroplasticity: A Single Case Study of Focal Hand Dystonia”) used 1 Hz repetitive transcranial magnetic stimulation (rTMS) targeting the left primary motor cortex (M1) of an individual diagnosed with focal hand dystonia. rTMS was applied over five daily thirty-minute sessions. Using a fine-grained kinematic analysis, they found that rTMS resulted in improved motor coordination, a finding that underlines the importance of adopting measures that are sufficiently sensitive to detect behavioral improvements.

Only recently, novel neurotechnological tools, such as brain/neural-machine interfaces (B/NMI) [10–14] or closed-loop brain and spinal cord stimulation [15], were developed that provide promising means to modulate CNS plasticity triggering neural recovery. A remarkable demonstration of these new targeted neurotechnologies was recently provided by Wagner et al. [16] demonstrating restoration of walking in individuals who sustained a spinal cord injury several years ago with permanent motor deficits despite extensive rehabilitation efforts. A few months of individualized spatiotemporal electrical stimulation of the lumbosacral spinal cord resulted in regained voluntary control over previously paralyzed muscles, even in the absence of stimulation.

As our understanding of the underlying mechanisms of neural recovery improves and neurotechnologies advance, more of such demonstrations will be ahead of us. We hope that this special issue will contribute towards such improved understanding of the relationship between neural plasticity and functional recovery and will give new impulses on how neurorehabilitation can be advanced through neurotechnological tools.
